# Characteristics of Gastroenteritis Outbreaks Investigated in Singapore: 2018–2021

**DOI:** 10.3390/ijerph21010064

**Published:** 2024-01-06

**Authors:** Muhd Tarmidzi Fua’di, Benjamin Er, Sylvester Lee, Pei Pei Chan, Joanna Khoo, Desmond Tan, Huilin Li, Imran Roshan Muhammad, Pream Raj, Lalitha Kurupatham, Vernon Lee, Li Kiang Tan, Joanne Sheot Harn Chan, Angela Li, Kyaw Thu Aung

**Affiliations:** 1 National Centre for Food Science, Singapore Food Agency, 7 International Business Park, Techquest, Singapore 609919, Singapore; muhd_tarmidzi_fuadi@sfa.gov.sg (M.T.F.); benjamin_er@sfa.gov.sg (B.E.); joanna_khoo@sfa.gov.sg (J.K.); desmond_tan@sfa.gov.sg (D.T.); li_huilin@sfa.gov.sg (H.L.); tan_li_kiang@sfa.gov.sg (L.K.T.); chan_sheot_harn@sfa.gov.sg (J.S.H.C.); angela_li@sfa.gov.sg (A.L.); 2 Communicable Disease Division, Ministry of Health, Singapore 169854, Singapore; sylvester_lee@moh.gov.sg (S.L.); chan_pei_pei@moh.gov.sg (P.P.C.); imran_roshan_muhammad@moh.gov.sg (I.R.M.); pream_raj@moh.gov.sg (P.R.); vernon_lee@moh.gov.sg (V.L.); 3 Department of Food Science and Technology, National University of Singapore, 2 Science Drive 2, Singapore 117543, Singapore; 4 School of Biological Sciences, Nanyang Technological University, 60 Nanyang Drive, Singapore 637551, Singapore

**Keywords:** gastroenteritis outbreaks, foodborne pathogens, sources of food, viral, norovirus, bacterial, *C. perfringens*, *Salmonella*, caterers, preschools

## Abstract

There is a need to study the characteristics of outbreaks via Singapore’s outbreak surveillance system to understand and identify the gaps in food safety for targeted policy interventions due to the increasing trend in gastroenteritis outbreaks and consequential increase in foodborne-related deaths and economic burden on public health systems worldwide. A total of 171 gastroenteritis outbreaks were investigated in Singapore from January 2018 to December 2021. This study analyzed the annual trend of investigated gastroenteritis outbreaks, the proportion of outbreaks by implicated sources of food, and the proportion of the type of pathogens identified from human cases, food samples, and environmental swabs collected from outbreak investigations. Among the foodborne gastroenteritis outbreaks (n = 121) investigated in Singapore, approximately 42.1% of the outbreaks had food prepared by caterers, 14.9% by restaurants, and 12.4% had food prepared by in-house kitchens. *Clostridium perfringens* and *Salmonella* were the most common causative pathogens in foodborne outbreaks throughout the analysis period. The food samples and environmental swabs collected were mostly detected for *Bacillus cereus*. Norovirus was the most common causative pathogen in non-foodborne outbreaks and was mainly attributable to preschools. This highlights the importance of monitoring and educating the catering industry and preschools to prevent future outbreaks.

## 1. Introduction

Gastroenteritis can be caused by the consumption of food contaminated with foodborne pathogens, which may occur at any stage from farm to fork [1]. This could be due to factors such as cross-contamination due to poor food safety and food handling practices during food production and preparation or the inadequate removal of naturally occurring pathogens via cooking [1]. Gastroenteritis can also be transmitted from person to person via direct contact or from fomites in the environment [1].

The World Health Organization reported that an estimated 600 million cases of gastroenteritis-related diseases were caused by foodborne pathogens in 2010. From this estimate, 420,000 deaths were associated with foodborne pathogens [2], which equates to 550 disability-adjusted life years (DALYs) per 100,000 population [3]. Gastroenteritis outbreaks caused by foodborne pathogens pose a significant health burden globally, especially on the vulnerable population. A third of deaths due to foodborne pathogens occur among children under 5 years of age, observed mainly in developing countries [4].

Trends in gastroenteritis outbreaks around the world are on the increase. A study in Korea observed an increase in the number of outbreaks by 42%, from 422 outbreaks in 2015 to 600 outbreaks in 2019 [5]. Another study in China also shared similar increasing trends of gastroenteritis outbreaks from 2013 to 2017 [6]. Based on the national disease surveillance statistics for Singapore, there was an increase in the number of food poisoning notifications from 4.8 to 7.5 per 100,000 population from 2013 to 2019 [7,8]. In 2020 and 2021, however, Singapore’s number of food poisoning notifications decreased to 3.9 and 4.7 per 100,000 population, respectively. Similar reductions were also observed in the UK and the US in 2020, which were likely due to COVID-19 control measures.

Studies overseas have shown an increase in economic costs due to gastroenteritis illnesses over the years. In the United States, it was reported that the economic cost had increased by USD 2 billion from 2013 to 2018 [9], while the World Bank reported an estimated annual cost of USD 95.2 billion and USD 15 billion in total productivity loss and cost of treating illnesses associated with gastroenteritis in low- and middle-income countries, respectively [10]. With the increasing trends in gastroenteritis outbreaks and consequential increase in foodborne-related deaths and economic burden on public health systems worldwide, there is a need to study the characteristics of outbreaks in Singapore via Singapore’s outbreak surveillance system for the prevention and control of gastroenteritis illnesses.

## 2. Materials and Methods

### 2.1. Study Design

Incidents of gastroenteritis illness in Singapore are reported to the Singapore Food Agency (SFA) via an online feedback portal, phone calls, or emails. Besides public feedback channels that receive feedback on alleged food poisoning incidents, the Singapore Ministry of Health (MOH) has disease surveillance systems in place where medical practitioners, laboratories, and identified institutions provide information on foodborne disease cases or gastroenteritis clusters. SFA and MOH, together with other relevant public agencies, jointly conduct outbreak investigations when notifications to incidents hit predefined triggers. A gastroenteritis outbreak is defined as two or more cases who developed symptoms of gastroenteritis (e.g., vomiting or diarrhea) and are linked to an institution or food establishment, in which epidemiological investigations suggest transmission occurred through (i) the consumption of food from the premises (i.e., foodborne) and/or (ii) human-to-human contact or contact with common surfaces within a defined period (i.e., non-foodborne). 

A total of 171 gastroenteritis outbreaks involving 7538 human cases were investigated in Singapore from January 2018 to December 2021. These gastroenteritis outbreaks were categorized as foodborne and non-foodborne (e.g., human-to-human transmission) causes based on epidemiological, clinical and microbiological evidence. For outbreaks with insufficient microbiological evidence, epidemiological evidence is used to infer the mode of transmission, where possible. During the outbreak investigation, stool samples of 1050 human cases were collected for laboratory identification of causative pathogens. In addition, a total of 1024 food samples were selected for the microbiological analysis based on the food consumption history of human cases (i.e., common food consumed) and epidemiological knowledge gathered from the outbreaks, while 917 environmental swabs were conducted based on kitchen operations via interviews with food handlers (i.e., high touch points or surfaces in contact with ready-to-eat foods within the implicated premises). This study conducted a retrospective analysis of the information gathered from investigated gastroenteritis outbreaks reported via SFA and MOH feedback and surveillance systems. Data from MOH’s outbreak investigation reports were extracted and compiled for analysis in this study.

### 2.2. Data Analysis

Data from the investigation reports include background information (e.g., the date of first notification of outbreak, outbreak setting, and the source of implicated food), epidemiological and clinical information (e.g., the number of human cases, attack rate, and symptoms experienced by cases) and microbiological findings (e.g., pathogens detected in stool samples, food samples, and environment swabs). Data on the source of implicated food were categorized into the various licensed premises types regulated by the SFA. These include caterers, restaurants, hawker stalls, in-house kitchens, canteens, and bakeries (as shown in Table 1). Stool samples were collected, where available, and submitted to the National Public Health Laboratory or hospital laboratories for identification of etiological agents in accordance with the clinical microbiology and diagnostics procedures. Food and environmental samples were collected from implicated premises using sterile bags and swabs. The samples were kept in a cooler bag with ice before being transported to SFA laboratories. The samples were tested using accredited microbiological methods that were accredited under the Singapore Accreditation Council Singapore Laboratory Accreditation (SAC-Singlas) Scheme [11] for pathogen detection and identification. The causative agent(s) of an outbreak were determined based on the pathogen(s) identified in the stool samples of cases, food samples, environmental swabs, and epidemiological findings. In certain outbreaks, the causative agent may be undetermined due to the lack of microbiological findings. The proportion of outbreaks by sources of food was tabulated by year to determine the top three settings in which outbreaks had occurred across time. The study calculated the percentage of gastroenteritis outbreaks by outbreak settings and foodborne pathogens relative to all gastroenteritis outbreaks by year. The median counts and IQR of *B. cereus* and *S. aureus* were also analyzed and provided in the results. The analysis and visualizations of the datasets were performed with Tableau (2019.2), Excel software (Microsoft version 2309) and R studio (v 1.4.1103).

### 2.3. Ethical Considerations

No ethics approval was required for this study as the analyses were carried out on data that were collected retrospectively as part of outbreak investigations. Additionally, only aggregated data without identifiable information, such as names of cases, were used in this study.

## 3. Results

**Figure 1:**
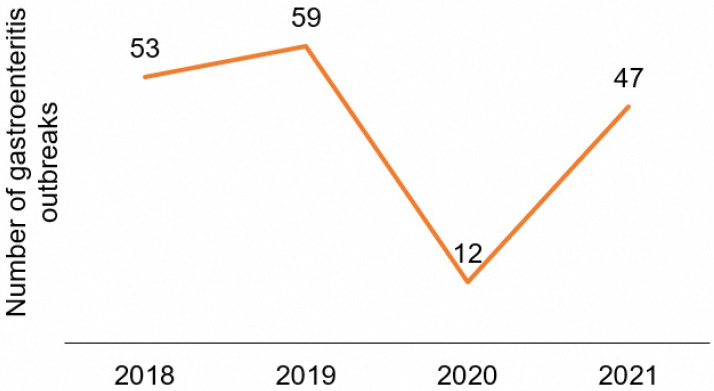
Number of gastroenteritis outbreaks investigated yearly (2018–2021).

A total of 171 gastroenteritis outbreaks of both foodborne and non-foodborne causes were investigated from January 2018 to December 2021. The mean number of outbreaks investigated annually is 43, with a range of 12 to 59, as shown in Figure 1. The highest number of outbreaks was observed in 2019 and the lowest in 2020.

**Figure 2:**
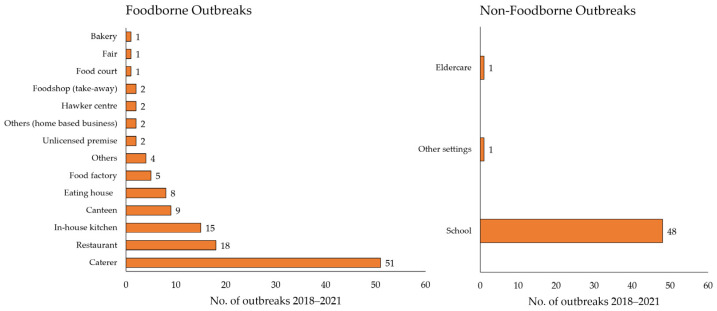
Cumulative number of gastroenteritis outbreaks investigated (2018–2021).

Figure 2 provides a breakdown of the cumulative number of gastroenteritis outbreaks investigated from 2018 to 2021 by setting. The top three sources of food implicated in foodborne outbreaks were caterers, restaurants, and in-house kitchens, while schools made up the majority of non-foodborne outbreaks. Of the 121 foodborne gastroenteritis outbreaks reported between 2018 and 2021, approximately 42.1% (n = 51) of the outbreaks had food prepared by the caterers (Table 1). Caterers also contributed to the greatest number of cases affected over the years. On the other hand, of the 50 non-foodborne gastroenteritis outbreaks, 96% (n = 48) were at schools.

As shown in Table 2, *C. perfringens*, *Salmonella* and norovirus were the most common causative pathogens in foodborne outbreaks throughout the analysis period. The other causative pathogens include pathogenic *E. coli* (non-Shiga toxin-producing *E. coli*), *V. parahaemolyticus*, *B. cereus*, and *S. aureus*. As shown in both Table 1 and Table 2, the main causative pathogen of non-foodborne outbreaks, which mainly involved preschools, was norovirus. Of the 171 outbreaks, 69.6% (119/171) were determined to be point source outbreaks. The two major pathogens that were identified to be the contributing factors were *C. perfringens* (16.8%, 20/119) and *Salmonella* (15.1%, 18/119).

A total of 1024 food samples were collected during gastroenteritis outbreak investigations from 2018 to 2021. Only 9.8% (100/1024) of the food samples collected were detected with pathogens. Table 3 shows the breakdown of pathogens found in food samples by year. The top 3 bacterial pathogens that were detected in food samples from 2018 to 2021 were *B. cereus* (43.9%), *Staphylococcus aureus* (13.3%), and *Escherichia coli* (12.2%). In total, 17.3% of all food samples collected were detected with more than one type of pathogen. The median counts for *B. cereus* and *S. aureus* detected in food samples were 2.4 log CFU/g (IQR 2.0–3.0 log CFU/g) and 3.1 log CFU/g (IQR 1.6–3.6 log CFU/g), respectively.

A total of 917 environmental swabs were collected from gastroenteritis outbreak investigations conducted from 2018 to 2021. Only 14.6% (134/917) of the environmental swabs collected were detected with pathogens. Table 4 shows the top 3 pathogens were *B. cereus* (7.0%), norovirus (4.6%), and *S*. Enteritidis (1.2%). The median counts for *B. cereus* and *S. aureus* detected in environmental swabs were 2.3 log CFU/g (IQR 2.0–2.7 log CFU/g) and 1.9 log CFU/g (IQR 1.0–2.7 log CFU/g), respectively.

## 4. Discussion

### 4.1. Control Measures in Response to COVID-19 Resulted in a Decline in Gastroenteritis Outbreaks in 2020

In 2020, the number of gastroenteritis outbreaks in Singapore was significantly lower compared to other years. This was likely due to the implementation of governmental measures to curb the spread of COVID-19, including a strict lockdown, i.e., Circuit Breaker, and the restriction on food establishments to offer only takeaway options. This trend was also observed in other countries, such as the United Kingdom and the United States, where the number of reported outbreaks was substantially lower for the duration of the COVID-19 response period as compared to previous periods [12]. The subsequent relaxation of COVID-19 measures in Singapore, such as the gradual reopening of schools, workplaces, and food establishments for dining-in (albeit in a smaller group size), saw the number of outbreaks rebounding back in 2021. The decline in specific foodborne diseases during COVID-19 was observed in several countries. For instance, a study conducted in Spain reported a decrease in norovirus incidence during the COVID-19 pandemic. A similar finding was observed in Australia, China, England, and the United States [13]. Another study conducted in the Netherlands reported a significant decrease in the incidence of human salmonellosis during the COVID-19 pandemic [14].

### 4.2. Caterers Accounted for a Substantial Proportion of Foodborne Outbreaks

A large proportion of outbreaks could be attributed to transmissions from food prepared by licensed caterers for events and gatherings and dining in at restaurants (refer to Table 1). Measures on gatherings in response to COVID-19 could have led to a decrease in engagement of caterers and dining out at restaurants, which ultimately resulted in a decrease in the incidence of foodborne outbreaks. These restrictions were also extended to schools, and home-based learning was implemented to reduce interaction among children, which in turn reduced the frequency of consumption of food prepared by caterers during school events. These findings indicate the importance of food providers, particularly caterers, ensuring that food is prepared for consumption in a safe way.

Based on the outbreak investigation findings in Singapore as described in this study, caterers have been shown to be commonly associated with gastroenteritis outbreaks, which was similarly observed in statistics from other countries like the United States, the Netherlands, and the United Kingdom [15]. This is due to the process chain of preparation to the serving of food, which can introduce foodborne pathogens via contamination due to poor personal hygiene, undercooking from cooking in bulk, prolonged storage of food at ambient temperatures, and infected food handlers [16]. These are potentiating factors for foodborne diseases due to *C. perfringens* and *Salmonella*, which were the most common causative pathogens of outbreaks occurring from 2018 to 2021. In 2011, the Centre for Disease Control and Prevention (CDC) reported that the top two ranked bacterial pathogens that caused gastroenteritis illnesses in the United States were *Salmonella* and *C. perfringens* [17]. In another recent study, it was reported that *C. perfringens* was second to *Salmonella* in the estimated annual number of cases due to bacterial agents in the United States [18]. This was similarly observed in Singapore, where *C. perfringens* and *Salmonella* were the top two causative pathogens of the outbreaks.

The increase in gastroenteritis outbreaks in the United States was likely attributed to the expanding catering industry due to the changing societal eating habits [19]. The common foods associated with *C. perfringens* include meat, poultry products, soups, sauces such as gravy, and other precooked food [20] and are typically associated with improper cooking or the inadequate heating of food products [21]. Caterers usually have to perform cooking in bulk to serve larger groups of customers, and therefore it is crucial for them to rapidly cool food products. This is due to the fact that when cooked food products are cooled, it passes the growth temperature range of *C. perfringens* (5 °C to 60 °C), and if the cooling times are extended with the inclusion of other favourable parameters (e.g., pH or water activity), it may support the growth of *C. perfringens* [22]. In order to prevent the risk of *C. perfringens*, bulk cooking should be avoided whenever possible, or food products should be cooked in smaller batches. When cooking large amounts of food together in a single batch, food after cooking should be placed in shallower containers and kept above the temperature danger zone to ensure food is not kept for long periods of time at temperatures under which *C. perfringens* proliferate rapidly [23,24].

For outbreaks caused by *Salmonella*, they are usually attributed to the consumption of contaminated food products that are usually from poultry, pork, and egg products [20]. However, they are not restricted to only these food products, as the CDC reported that other strains of *Salmonella* species were also detected in other food products (e.g., onions, shrimp, and peaches) [25]. Food handlers are a likely medium for *Salmonella* transmission as they are in direct contact with food reservoirs within the process chain. There is a higher risk of cross-contamination if a food handler prepares raw food and cooks food simultaneously. The risk of contamination could be prevented by practicing good food hygiene practices (e.g., washing hands with soap and water after handling raw food, the use of separate equipment and utensils for raw and cooked food, and ensuring that raw food and cooked food are stored separately) [21].

Outbreaks in which pathogens were not detected in cases, food samples, and environmental swabs did not reveal causative agents; hence, their contributing factors could not be determined. Although the causative agents of these outbreaks could not be determined, many of these outbreaks had findings of poor personal hygiene, environmental hygiene and/or poor food preparation practices. There is a need for the food sector to strengthen its food process chain and ensure that food is cooked, cooled thoroughly, and stored in proper conditions prior to consumption in order to prevent foodborne outbreaks. In June 2014, the Singapore Food Agency (SFA) mandated the implementation of food safety management systems (FSMS) in all licensed food catering services establishments (which later extended to all licences with permission to provide catering as an ancillary service in April 2019) as a systematic approach to improve food safety [26]. While it had been demonstrated that legally mandated FSMS for caterers had been associated with a significant reduction in foodborne disease outbreaks [26], more could be done by the food companies to validate production and process controls since the main foodborne causative pathogens identified (*C. perfringens* and *Salmonella*) were commonly associated with failures in food preparation practices (e.g., improper heating and cooling of food) and to incorporate learning points in periodic trainings as a reminder for food handlers to adhere to good hygiene practices. In addition, fostering a positive food safety culture within the food companies will further enhance the food safety practices and behaviour of food handlers. Food safety culture is defined as the shared attitudes, values, and beliefs towards hygienic behaviour used within a food handling environment [27]. Companies that focus on creating a positive food safety culture are more likely to motivate workers to engage in food safety practices [27]. More could be done to encourage food companies to adopt a positive food safety culture to reduce the risk of foodborne illness.

### 4.3. Increasing Number of Norovirus Outbreaks in Preschools

As shown in Table 1 and Table 2, the study observed a sizeable proportion of non-foodborne outbreaks occurring, particularly in the preschool setting in 2019 and 2021. It has been established that norovirus is primarily transmitted via the fecal–oral route, via direct contact with infected persons, environmental surfaces contaminated with vomitus or feces, or contaminated food or water [28]. Although norovirus was not detected in food samples collected from the investigations of these outbreaks, the environmental swabs that were collected tested positive for the virus in addition to human stool samples. This suggests that human-to-human transmission may play an important role in the transmission of norovirus.

Preschools are common settings for norovirus viral gastroenteritis outbreaks, and there could be an interplay of factors, e.g., personal hygiene of students, size and ventilation of classrooms, and adherence to disinfection practices upon a vomiting incident, which could have led to the outbreak. The detection of norovirus on environmental swabs in our study, as well as hygiene investigation findings, suggest that these preschools lack adherence to recommended cleaning guidelines (e.g., the use of non-chlorine instead of chlorine-based disinfectants, high-contact surfaces not thoroughly cleaned, and the cross-contamination of cleaning equipment). From our study, a third of preschools implicated in norovirus outbreaks did not adhere to recommended disinfection guidelines. The indoor environmental conditions of preschools, such as classroom segregation and the availability of proper ventilation, likely also play a role in the transmission dynamics of norovirus, which can also be transmitted by aerosolized particles from vomitus [29]. In addition, the increase in norovirus outbreaks in preschools in 2021 could also be due to “immunity debt”, which may have been acquired due to a lack of exposure to norovirus in 2020 due to stricter COVID-19 control measures then, which was similarly observed in the United Kingdom [30].

The observable proportion of norovirus-related outbreaks observed during the study period highlights the importance of adopting preventive measures to mitigate the impact of norovirus on vulnerable preschool-going children. Awareness amongst educators and parents of preschool children needs to be raised, particularly on ensuring good personal hygiene practices among preschool staff and children and proper cleaning and disinfection of preschool premises. Staff and children who are unwell with gastroenteritis should remain at home and return to school only when fully recovered to minimize the risk of transmission of norovirus in the preschools. Preschools must also properly screen the children for signs and symptoms of gastroenteritis prior to entry for early identification and isolation of cases. The implementation of good personal hygiene practices and increasing awareness of norovirus transmission at preschools will help decrease the incidence of other infectious diseases as well.

### 4.4. Detection of Bacillus cereus in Food and Environmental Samples Highlights the Importance of Proper Food Safety Practices

As shown in Table 3 and Table 4, *Bacillus cereus* was the most common pathogen detected in food samples and environmental swabs collected during gastroenteritis outbreak investigations conducted from 2018 to 2021. As *B. cereus* is ubiquitous in the environment such as soil, it can be inherently found in a wide range of food products, especially for the products that are of plant origins. *B. cereus* can proliferate and form toxins in foods when such food items are subjected to time-temperature abuse [31].

To prevent the growth of *B. cereus*, it is therefore essential for food handlers to practice proper food handling, such as cooking food thoroughly to its safe internal temperature and ensuring that cooked food is kept within the safe temperature range above 60 °C for hot foods before serving and less than 5 °C for cold food if food is served later [32]. Mitigation measures such as implementing stricter food hygiene protocols and the re-training of food handlers would help to improve overall food hygiene and handling standards and consequently reduce the number of pathogens found in food and the environment. This would ultimately reduce the risk of gastroenteritis outbreaks in Singapore. In addition to proper hygiene practices and disinfection, proper temperature control (i.e., refrigeration) of cooked food would help limit the growth of *B. cereus* and its spores [33].

A study conducted in the Netherlands also detected *B. cereus* in the majority of food samples collected (e.g., cereals, pasta, rice) from their past year’s surveillance data where the presence of *B. cereus* in food samples was due to improper temperature management and contamination due to poor hygiene and improper cleaning [34]. Another study conducted in Italy highlighted that *B. cereus* was detected in environmental swabs from various cooking equipment [35]. Similarly, findings from our study observed that *B. cereus* was detected in a diverse variety of food samples (e.g., ready-to-eat fish, chicken, milk powder, vegetables, rice/noodles, and sauces) and environmental swabs (e.g., knife, chopping board, utensils, crockeries, preparation table, door handle, and kitchen appliances). Thus, it is important to ensure good temperature control, proper food handling practices, and the routine disinfection of food contact surfaces during food preparation, as once the food is contaminated with *B. cereus*, cooking may kill the bacteria but not remove the toxins that were produced [36].

## 5. Strengths and Limitations

To the best of our knowledge, this is the first report on the characteristics of gastroenteritis outbreaks investigated in Singapore between the years 2018 and 2021. This adds to the paucity of literature for evaluating the trends of gastroenteritis outbreaks in Singapore. Nonetheless, there were several limitations to the study. Firstly, the number of gastroenteritis outbreaks in Singapore studied was likely an underrepresentation of the actual number of outbreaks that had occurred from 2018 to 2021, as not all outbreaks are reported to the authorities. Secondly, the reporting time lag following the symptom onsets of cases and subsequently delay in sample collection (clinical, food, and environmental) makes it difficult for investigations to ascertain the extent of outbreaks, source of infection and mode of transmission, contributing to the underrepresentation of the actual number of outbreaks.

## 6. Conclusions

Our study showed that the incidence of gastroenteritis outbreaks that were serious enough to trigger investigations was kept relatively low while throughout the study period. The decline in the number of gastroenteritis outbreaks in the year 2020 was likely due to COVID-19 restriction measures on social gatherings. *C. perfringens* and *Salmonella* were identified to be the main causative agents for foodborne gastroenteritis outbreaks, and the majority of these outbreaks involved caterers.

The study observed a sizeable proportion of non-foodborne outbreaks occurring, particularly in the preschool setting. To strengthen preventive measures, further studies can be conducted to identify specific risk factors associated with norovirus outbreaks in preschools. Outbreak prevention and control in these settings should focus on handwashing, cleaning, and disinfection with effective products and the exclusion of ill children and staff. Appropriate awareness of norovirus transmissions in preschools should be enhanced via advisories and engagement with parents and staff.

The detection of *B. cereus* in food samples and environmental swabs shows the importance of practicing proper food safety practices. This can be achieved by ensuring good temperature control, proper food handling practices, and the implementation of routine disinfection of food contact surfaces. Proper temperature control of cooked food, such as refrigeration and hot holding, is especially important in limiting the growth of *B. cereus* and its spores. These proper food safety practices would help improve overall food hygiene and handling standards and consequently reduce the number of pathogens found in food and the environment.

The findings in the study may be used to guide food authorities and companies in the development of targeted policy interventions and programmes for the prevention and control of gastroenteritis illnesses.

## Figures and Tables

**Table 1:** Proportion of gastroenteritis outbreaks investigated by setting, from 2018 to 2021. Ranked by alphabetical order.

Gastroenteritis Outbreaks	2018 (n = 53)		2019 (n = 59)		2020 (n = 12)		2021 (n = 47)	
Foodborne	No. of Outbreaks (%)	No. of Cases	No. of Outbreaks (%)	No. of Cases	No. of Outbreaks (%)	No. of Cases	No. of Outbreaks (%)	No. of Cases
Bakery	0	0	1 (1.7)	24	0	0	0	0
Canteen	2 (3.8)	105	4 (6.8)	136	1 (8.3)	31	2 (4.3)	156
Caterer	27 (50.9)	1213	14 (23.7)	680	3 (25.0)	241	7 (14.9)	510
Eating house *	0	0	3 (5.1)	20	2 (16.7)	40	3 (6.4)	108
Fair	1 (1.9)	150	0	0	0	0	0	0
Food court	1 (1.9)	32	0	0	0	0	0	0
Food factory	0	0	2 (3.4)	75	1 (8.3)	35	2 (4.3)	89
Foodshop (take-away)	0	0	0	0	1 (8.3)	5	1 (2.1)	9
Hawker centre ^#^	1 (1.9)	4	0	0	0	0	1 (2.1)	52
Home-based business	0	0	1 (1.7)	35	0	0	1 (2.1)	72
In-house kitchen	4 (7.5)	185	7 (11.9)	215	2 (16.7)	115	2 (4.3)	116
Others	1 (1.9)	80	0	0	0	0	3 (6.3)	130
Restaurant	9 (17.0)	331	4 (6.8)	91	1 (8.3)	30	4 (8.5)	263
Unlicensed premises	0	0	1 (1.7)	23	0	0	1 (2.1)	70
**Non-foodborne**								
Eldercare	0	0	1 (1.7)	30	0	0	0	0
Other settings	1 (1.9)	282	0	0	0	0	0	0
School	6 (11.3)	151	21 (35.6)	947	1 (8.3)	23	20 (42.6)	634
Preschools	3 (50.0)	91	15 (71.4)	421	1 (100.0)	23	20 (100.0)	634
Non-preschools ^^^	3 (50.0)	60	6 (28.6)	526	0	0	0	0

^^^ Includes primary schools, secondary schools, and tertiary institutes. * Eating house refers to an open-air premises that has various food stalls. ^#^ Hawker centre refers to an open-air complex that houses many food stalls that sell a wide variety of food and are mostly located at the heart of housing estates.

**Table 2:** Proportion of outbreaks by specific type of causative pathogen, ranked by number of outbreaks from 2018 to 2021.

Causative Pathogen of Outbreak	2018	2019	2020	2021
	No. of Outbreaks (%)	No. of Outbreaks (%)	No. of Outbreaks (%)	No. of Outbreaks (%)
**Foodborne Outbreaks**	20	18	7	13
**Bacterial pathogen**				
*C. perfringens*	10 (18.9)	3 (5.1)	2 (16.7)	5 (10.6)
*Salmonella* spp.	4 ^a^ (7.5)	7 ^b^ (11.9)	3 ^c^ (25.0)	4 ^d^ (8.5)
Pathogenic *E. coli*	0	3 ^e^ (5.1)	0	0
*V. parahaemolyticus*	0	0	0	2 (4.3)
*B. cereus*	1 (1.9)	0	0	1 (2.1)
*S. aureus*	1 (1.9)	1 (1.7)	0	0
**Viral pathogen**				
Norovirus	4 (7.5)	4 (6.8)	2 (16.7)	1 (2.1)
**Non-foodborne Outbreaks**	5	19	0	18
**Viral pathogen**				
Norovirus ^~^	5 (9.4)	17 (28.8)	0	18 (38.3)
Rotavirus	0	2 (3.4)	0	0
**Causative pathogen not established**	28 (52.8)	22 (37.3)	5 (41.7)	16 (34.0)
Total	53	59	12	47

^a^ *Salmonella* Enteritidis (1), *Salmonella* Weltevreden (1), *Salmonella* Typhimurium (1), ^b^ *Salmonella* Enteritidis (6), ^c^ *Salmonella* Enteritidis (2), ^d^ *Salmonella* Enteritidis (2), *Salmonella* Kirkee (1), *Salmonella* Paratyphi (1). ^e^ Includes Enteropathogenic *E. coli* (1), Enterotoxigenic *E. coli* (1), and Enteropathogenic *E. coli* and Enteroaggregative *E. coli* (1). ^~^ Breakdown of norovirus outbreak associated in preschools: 2018 (2/3), 2019 (12/15), 2021 (18/20).

**Table 3:** Breakdown of specific pathogens identified in food samples collected during outbreak investigations from 2018 to 2021.

Pathogens Detected in Food Samples	No. of Food Samples Detected with Pathogens (%)			
	2018	2019	2020	2021
**Bacterial**	27 (9.1)	33 (9.9)	7 (12.1)	31 (9.3)
*B. cereus*	14 (4.7)	12 (3.6)	4 (6.9)	13 (3.9)
*S. aureus*	3 (1.0)	7 (2.1)	0	3 (0.9)
*E. coli* **	2 (0.7)	6 (1.8)	1 (1.7)	3 (0.9)
*S.* Enteritidis	0	1 (0.3)	0	3 (0.9)
*S.* Typhimurium	3 (1.0)	0	0	0
*V. parahaemolyticus*	0	0	1 (1.7)	0
*V. cholerae*	0	0	0	1 (0.3)
*S.* Albany	1 (0.3)	0	0	0
*C. perfringens*	0	0	0	1 (0.3)
*C. jejuni*	0	0	0	1 (0.3)
*Campylobacter* spp.	1 (0.3)	0	0	0
*B. cereus*, *E. coli* ** ^a^	1 (0.3)	2 (0.6)	0	2 (0.6)
*E. coli* **, *S. aureus* ^a^	1 (0.3)	1 (0.3)	1 (1.7)	0
*B. cereus*, *S. aureus* ^a^	0	2 (0.6)	0	0
*E. coli* **, *B. cereus*, *S. aureus* ^a^	0	2 (0.6)	0	0
*V. cholerae*, *V. parahaemolyticus* ^a^	0	0	0	2 (0.6)
*B. cereus*, *S.* Typhimurium ^a^	1 (0.3)	0	0	0
*E. coli* **, *C. perfringens*	0	0	0	1 (0.3)
*V. cholerae*, *V. parahaemolyticus*, *V. vulnificus* ^a^	0	0	0	1 (0.3)
**Viral**	1 (0.3)	1 (0.3)	0	0
Norovirus	1 (0.3)	1 (0.3)	0	0
**Food samples not detected with pathogens**	269 (90.6)	300 (89.8)	51 (87.9)	304 (90.7)
**Total**	297	334	58	335

^a^ Multiple pathogens detected in food samples. ** *E. coli* strains detected that appeared to be non-pathogenic.

**Table 4:** Breakdown of specific pathogens identified in environmental swabs collected during outbreak investigations from 2018 to 2021.

Environmental Swabs Detected with Pathogens	No. of Environmental Swabs Detected with Pathogens (%)			
	2018	2019	2020	2021
**Bacterial**	24 (11.8)	38 (9.8)	6 (9.8)	23 (8.7)
*B. cereus*	19 (9.4)	31 (8.0)	5 (8.2)	9 (3.4)
*S.* Enteritidis	1 (0.5)	4 (1.0)	0	6 (2.3)
*S. aureus*	1 (0.5)	2 (0.5)	0	3 (1.1)
*E. coli* **	1 (0.5)	0	1 (1.6)	3 (1.1)
*S.* Typhimurium	2 (1.0)	0	0	0
*Campylobacter* spp.	0	0	0	1 (0.4)
*E. coli* **, *S. aureus* ^a^	0	0	0	1 (0.4)
*B. cereus*, *E. coli* ** ^a^	0	1 (0.3)	0	0
**Viral**	24 (11.8)	15 (3.9)	0	3 (1.1)
Norovirus	24 (11.8)	15 (3.9)	0	3 (1.1)
**Bacterial and Viral**	1 (0.5)	0	0	0
*B. cereus*, Norovirus ^a^	1 (0.5)	0	0	0
**Environmental swabs not detected with pathogens**	154 (75.9)	336 (86.4)	55 (90.2)	238 (90.2)
**Total**	203	389	61	264

^a^ Multiple pathogens detected in environmental swabs. ** *E. coli* strains detected that appeared to be non-pathogenic.

## Data Availability

The data presented in this study are confidential and not available upon request.
